# Effects of Cognitive Appraisals on Perceived Self-Efficacy and Distress during the COVID-19 Lockdown: An Empirical Analysis Based on Structural Equation Modeling

**DOI:** 10.3390/ijerph20075294

**Published:** 2023-03-28

**Authors:** Pierluigi Diotaiuti, Giuseppe Valente, Stefania Mancone, Stefano Corrado, Fernando Bellizzi, Lavinia Falese, Elisa Langiano, Guilherme Torres Vilarino, Alexandro Andrade

**Affiliations:** 1 Department of Human Sciences, Society and Health, University of Cassino and Southern Lazio, 03043 Cassino, Italy; 2 Department of Human Movement Science, Santa Catarina State University, Florianòpolis 88035-901, Brazil

**Keywords:** COVID-19 lockdown, worries, threat appraisal, growth appraisals, self-efficacy, distress, adjustment, SEM

## Abstract

During the COVID-19 lockdown, individuals and households had to responsibly manage the difficulties and problems caused by the restrictions on their mobility, such as the interruptions to work schedules, insecure food supplies, and the procurement of services and health care. The perceptions of risk as well as the fear of disease were strongly linked to worry, defined as a sequence of thoughts that evoke negative emotions and elevated levels of anxiety and distress. This study evaluated how different cognitive appraisals of an individual’s sources of worry could influence their perceived self-efficacy and directly or indirectly moderate their perceived general distress. A sample of 544 participants completed a survey that included questions based on the generalized self-efficacy scale, the sources of concern during the epidemic, the cognitive appraisal scale in emergency, and perceived discomfort. Subsequently, a structural-equation-modeling (SEM) analysis was performed to test the moderating role of cognitive appraisals and perceived self-efficacy on participants’ overall distress during a lockdown. Overall, the model reported acceptable fit values and confirmed the hypotheses of the study. An individual’s worries activated either a dysfunctional threat appraisal, which prompted a fear-and-closure response that then increased their overall state of distress; or two other functional appraisals (i.e., adaptive or supportive) that contributed to significantly improving the individual’s self-efficacy. Higher self-efficacy was shown to be associated with lower levels of perceived distress. For the purposes of prevention and distress containment, it would be appropriate to promote psycho-educational interventions that promote the adoption of appraisal strategies that are functional and beneficial for perceived self-efficacy.

## 1. Introduction

The COVID-19 pandemic and the related lockdowns led to an explosion of new research and data collection that further investigated the effects of specific restrictive measures adopted by different countries, on psychological health [1,2,3,4]. Recent studies have found that negative effects on mental health were particularly evident among psychiatric patients [5], individuals with previously diagnosed post-traumatic stress disorder [6], and COVID-19 patients [7], all of whom reported higher levels of anxiety, depression, and stress, as compared to healthy controls. Further research reported that restrictive measures forced individuals to modify their lifestyles; their risk perceptions; their confidence in others and in institutions; their sense of community; and their perceived self-efficacy [8,9]. Female gender, younger age, occupational status (employed), lower perceived wellbeing, and health risk factors and diseases, such as cancer, were predictors of mental health symptoms that were correlated with the COVID-19 pandemic and restrictive measures for public safety [10,11,12].

Extraordinary isolation and quarantine measures suddenly imposed drastic changes and limitations on the lifestyles and established routines of the general population. Individual and households had to responsibly manage the difficulties and problems caused by the restrictions on their mobility, such as interrupted work schedules, insecure food supplies, and the procurement of services and health care.

The perceptions of risk as well as the fear of disease have been strongly linked to worry, which is defined as a sequence of uncontrolled thoughts that evoke negative emotions and elevated levels of anxiety and distress related to fears about uncertainties and potential negative outcomes [13,14]. Triggered by anxious thoughts as well as by environmental events, worry is considered an apprehensive expectation about real-life issues such as health, relations, finances, work, and so on. On the continuum from normal to pathological, the severity of worry is determined by the frequency of uncontrollable, pervasive, and excessive thoughts, with high levels of worry being associated with negative health outcomes and somatic health complaints [15,16,17].

Within the set of an individual’s thoughts of concern, the source of concern towards which their attention is predominantly directed should also be considered, such as whether the source is internal (e.g., anxiety, physical tension, fear, boredom, sense of isolation and vulnerability) or external (e.g., limitations of economic resources and personal assets, etc.). A predominantly internal or external source of concern, with a related predominantly internal vs. external focus, is likely to influence the management and the perception of personal control over it [18].

Some scholars, referring to the theory of resource conservation [19,20], have argued that people with greater self-efficacy were able to acquire more resources to manage situations of actual or potential loss, as well as their consequences in the future [21]. Other studies have long confirmed the protective role of self-efficacy in stressful situations [22]. For example, transversal studies have demonstrated the medium-to-large effects of self-efficacy on the general discomfort, severity, and frequency of the symptoms of post-traumatic stress disorder (PTSD), while longitudinal studies have shown larger effects on the general discomfort and severity of PTSD symptoms, as self-efficacy was also related to improved somatic health (self-reported symptoms, including less pain, fatigue, and disability) [23,24].

With reference to SARS and other infectious diseases, further research has stressed the need, especially in European countries, to take action to increase self-efficacy, because low self-efficacy has led to a lack of motivation for protection [25]. Regarding the COVID-19 pandemic, recent studies have stressed the protective role of self-efficacy in limiting anxiety, the perception of fatigue in healthcare workers, and the signs of post-traumatic stress disorder [26,27,28,29,30,31,32].

In other subgroups of the general population, the moderating role of self-efficacy on the psychological well-being of college students and adolescents when managing their fears about COVID-19 has also emerged [33,34], as well as an inverse association between psychological distress and perceived self-efficacy in people who were pregnant during the pandemic [35]. A recent study using a large population sample confirmed that self-efficacy acted as a mediator in reducing perceived COVID-19-related stress, and in conjunction with its direct effects, self-efficacy partially mediated all other COVID-19-related beliefs (perceptions of disruption, health importance, and response effectiveness). Overall, it was determined that boosting self-efficacy was the most crucial component of resilience against experiencing high levels of stress [36].

Based on Bandura’s social cognitive theory that defined self-efficacy as one’s perceived ability to perform a target behavior, evidence has shown that higher self-efficacy has been linked to taking preventive measures during pandemic (e.g., handwashing, respiratory hygiene, wearing a mask when having symptoms). A major source of compliance and adherence to prescribed measures was related to having a sense of self-efficacy, as it promoted being amendable to change and adopting protective behaviors. Moreover, fear had minimal impact on people who had a sense of autonomy, which encouraged their fearless compliance [37,38,39]. Within the context of adolescents, their perceived abilities (self-efficacy) proved to be the strongest predictors of protective behaviors, suggesting that strategies to promote self-efficacy in adolescents should be carefully considered in order to assist them in enhancing their protective behaviors [40]. Research on the general population has also reported a simultaneous mediating effect co-produced by avoidance-based coping and self-efficacy, this may indicate an interactive relationship or interaction between these two psychological constructs that may influence a person’s perceived capability of adhering to COVID-19 precautionary measures [41].

Scholars have pointed out that cognitive appraisal may play an important role in the individual problem orientation of COVID-19 disruptions [42,43,44,45]. The influential transactional theory of stress and coping developed by Lazarus and Folkman contended that an individual’s assessment of a stressor significantly affected how they later coped with stressful situations [46]. Challenge appraisals have been linked to a perceived future benefit and show an adaptive response to stress in which the person perceives their situation as an opportunity for potential improvement and growth. Threat appraisals, on the other hand, have been linked to a perceived future loss or harm and are a sign of a maladaptive approach to stress in which the person emphasizes the situation’s potentially negative implications [47,48]. Challenge and threat appraisals have also been closely related to problem orientation, that is, an individual’s cognitive schemas that describe their thoughts and feelings about a particular problem, as well as their own ability to overcome the problem. People who framed problems cognitively as positive “opportunities” were more likely to use adaptive coping strategies than those who framed problems as “threats” [49]. When the relationship between stress appraisals and negative experiences during the pandemic was investigated, threat appraisals were associated with higher negative effects and more frequent stressors [42,50,51].

In considering the distinctiveness between maladaptive and adaptive approaches to difficulties, it has been helpful to consider the theory of protective motivation (PMT), which explicitly distinguishes between threat and coping appraisals, as concerning the multidimensional determinants of motivation [52]. Threat appraisal combines the perceived severity (perceptions about the severity of the harm) and perceived vulnerability (perceptions about likelihood of the harm) of a situation, without considering the perceived rewards (the potential positive outcomes). Coping appraisal is the sum of response efficacy (the perceived effectiveness of the recommended behavior in removing or preventing potential harm) and self-efficacy (the perception of an individual’s ability to successfully carry out the recommended behavior), less the response costs (the perceived or actual costs resulting from engaging in the recommended behavior) [53]. This model has also been used in recent studies focused on assessing predictors of COVID-19-related preventive behaviors [54,55,56].

Threat appraisal in a lockdown situation was linked to a maladaptive fear-oriented pattern, which is characterized by avoidant closure, increased inflexibility of behaviors and thoughts, and a tendency for social withdrawal. Coping appraisal, on the other hand, has been differentiated into two further adaptive patterns: the first is an approach-oriented coping strategy that focuses on increasing one’s knowledge of the problematic scenario in order to adopt functional ways of adapting to the new situation; and the second is growth-oriented and focuses on remaining open towards and actively supporting others in a similar situation while viewing the circumstances as an opportunity for personal growth and development. Thus, it could be said that these appraisal patterns activate an individual’s avoidance motivation and two different approach motivations for the situation, respectively.

On the basis of these arguments, the hypotheses that guided the implementation of the present study were the following:

1. Prevalence of internal concerns (e.g., anxiety, tension, fear, boredom, sense of isolation and vulnerability) and external concerns (e.g., general economic crisis, limitations of economic resources and personal assets) during difficult lockdown conditions could produce differential effects on the activation of the three identified appraisal patterns (i.e., fear-oriented; awareness and learning-oriented; growth-oriented);
2. Activated appraisal patterns could have differential effects on perceptions of self-efficacy;
3. Higher self-efficacy could be associated with a lower level of perceived distress during COVID-19 lockdowns.

**Figure 1:**
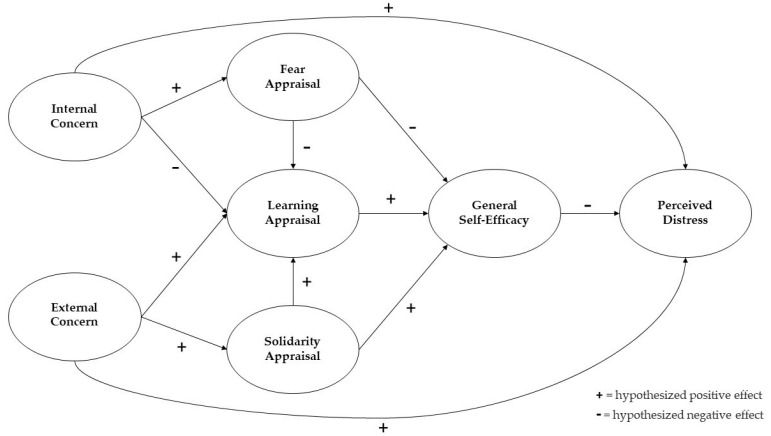
Hypothesized model.

These hypotheses were tested by a general path analysis model and by a sample of residents in the central/southern regions of Italy. Figure 1 shows the proposed conceptual model; based on a direct relationship between an individual’s concerns and perceived distress, the moderating roles of both types of appraisals and the individual’s perceived self-efficacy during lockdown conditions were highlighted.

## 2. Materials and Methods

### 2.1. Participants

The information needed to test the working hypotheses was gathered by administering a questionnaire to participants living in central and southern Italy. Students from a local university were involved in a representative proportion (at least 30%) of the 3 main regions of origin (Lazio, Campania, Molise). Each student was asked to invite (by forwarding an email requesting participation) at least 3 family members and/or friends in the age ranges of 18–38; 39–59; and >60. Therefore, those participants received an email inviting them to freely join the research by answering an online questionnaire. Data collection began on 5 April and ended on 24 May 2020. The Institutional Review Board of the University of Cassino and Southern Lazio approved the protocol. Participants were assured of their anonymity and the exclusive use of aggregated data for research purposes. The average completion time was approximately 15 minutes. In accordance with the Declaration of Helsinki, the administration of tools occurred following the release and signature of the form of informed consent to participate. A total of 544 participants completed the online questionnaire. The characteristics of the participants are shown in Table 1.

### 2.2. Measures

We designed an anonymous online survey to evaluated our hypotheses. When participants clicked on the link, the first page opened and included a cover letter that explained the goals and the procedures of the study and an informed consent statement. After providing their informed consent, the participants first provided socio-demographic data; current residential location; health data such as the presence of chronic pathologies and the presence of other people-at-risk in the household; regular activities in the household; and sports practices in the household.

The survey then investigated the following: (a) The predominant sources of concern during lockdown evaluated through the following 9 items on a 5-point Likert scale (from 1 = not at all, to 5 = very much): “How concerned are you about being without basic necessities?”; “How concerned are you about a possible national economic crisis?”; “How concerned are you about your economic resources?”; “How concerned are you about your future plans?”; “How concerned are you about your psychological well-being?”; “How anxious do you feel in this period?”; “How much has boredom increased in this period?”; “How pessimistic do you feel about the current situation?”; and “How much tension has increased at home during this period?”. The items were subjected to a confirmatory factor analysis (CFA) that reported based on a 2-factor structure and good-fit indices: χ^2^ (28) = 70.824; CFI = 0.971; TLI = 0.953; RMSEA = 0.053; RMSEA 90% C.I. (0.038; 0.069); and *p*-value = 0.350. These nine items were grouped according to two factors: internal concerns (five items referring to a person’s anxiety, boredom, pessimism, tension, mental health), and external concerns (four items referring to basic necessities, general economic crisis, personal economic resources, future plans). The tool showed good overall internal consistency: internal concerns: Cronbach’s α = 0.74 (CIs 95% 0.707; 0.776); McDonald’s ω = 0.75; (CIs 95% 0.717; 0.782); and external concerns: Cronbach’s α = 0.74 (CIs 95% 0.726; 0.791); and McDonald’s ω = 0.71; (CIs 95% 0.737; 0.807).

(b) The individual’s appraisal patterns were considered, including both threat and coping appraisals. Based on the Senninger’s tripartite model of fear, learning, and growth zones [57], a total of 12 items were chosen to represent a maladaptive appraisal oriented toward fear and closure, a functional appraisal oriented toward awareness and learning, and a functional appraisal oriented toward growth and supportive openness towards others. Specifically, the items were rated on a 5-point Likert scale (from 1 = not at all, to 5 = very much), as follows: “I try to find a purpose during the day”; “I identify my emotions”; “I am aware of the situation and think about how to behave”; “I live in the present and focus on the future”; “I look for a way to adapt to new changes”; “I let myself be infected by the fear and anger of others”; “I often get irritated”; “I easily get irritated”; “I think of others and look for ways to help them”; “I am empathetic to myself and to others”; “I make myself available to those in need”; and “I maintain a positive emotional state and instill hope in those around me”. Participants were asked to respond by indicating their level of agreement with each statement referring to the current lockdown. The items were then subjected to a confirmatory factor analysis (CFA) that reported based on a 3-factor structure and good-fit indices: χ^2^ (43) = 101.504; CFI = 0.974; TLI = 0.960; RMSEA = 0.050; RMSEA 90% C.I. (0.038; 0.063); and *p*-value = 0.478. These twelve items were therefore grouped into three factors: fear/closure-oriented appraisal (3 items), learning/awareness-oriented appraisal (5 items); solidarity/openness-oriented appraisal (4 items). The tool showed good overall internal consistency: fear/closure: Cronbach’s α = 0.82 (CIs 95% 0.787; 0.837); McDonald’s ω = 0.88; (CIs 95% 0.864; 0.898); learning/awareness: Cronbach’s α = 0.74 (CIs 95% 0.701; 0.771); McDonald’s ω = 0.74; (CIs 95% 0.703; 0.772); and solidarity/openness: Cronbach’s α = 0.70 (CIs 95% 0.653; 0.738); McDonald’s ω = 0.71; (CIs 95% 0.669; 0.746).

(c) Perceived self-efficacy in participants was considered. For this purpose, the generalized self-efficacy scale (GSE) [58] was administered in order to assess coping ability with daily inconveniences, as well as adaptation after experiencing a variety of stressful life events. The tool consisted of 10 items on a 4-point Likert scale, ranging from 1 (completely false) to 4 (completely true). The scale referred to the personal agency, namely the belief that individual actions were responsible for successful results, and contained items such as: “I remain calm in facing difficulties because I can rely on my own abilities to deal with them”; and “No matter what may happen to me, I can usually handle it”. The related CFA confirmed the single-factor structure of the instrument by reporting good-fit indices: χ^2^ (33) = 90.992; CFI = 0.972; TLI = 0.962; RMSEA = 0.057; RMSEA 90% C.I. (0.043; 0.071); and *p*-value = 0.196. The reliability assessment also showed adequate values: Cronbach’s α = 0.88 (CIs 95% 0.857; 0.889); and McDonald’s ω = 0.88; (CIs 95% 0.863; 0.893).

(d) Perceived distress measured 5 items according to a 5-point Likert scale, ranging from 1 (not at all) to 4 (very much): “How difficult is it for you to accept these restriction provisions?”; “How much has your vision of the future worsened in this period of lockdown?”; “How much has your stress level increased in this period of lockdown?”; “How much does this actual change in habits upset you?”; “Do you think the government’s current restrictive measures are appropriate?” (reverse item); and “How concerned are you that the current restrictions will last for a longer period than expected?”. The related CFA confirmed the single-factor structure of the instrument by reporting good-fit indices: χ^2^ (5) = 10.528; CFI = 0.992; TLI = 0.985; RMSEA = 0.045; RMSEA 90% C.I. (0.00; 0.083); and *p*-value = 0.524. The reliability assessment also showed adequate values: Cronbach’s α = 0.75 (CIs 95% 0.712; 0.777); and McDonald’s ω = 0.77; (CIs 95% 0.743; 0.802).

## 3. Statistical Analysis

The data were processed using the statistical software SPSS version 22 and Amos IBM version 22. The analyses performed were a descriptive statistics that illustrated the socio-demographic information; Pearson and Spearman’s bivariate correlations for all major measures of self-efficacy, concerns, and cognitive appraisals; perceived distress (significant at *p* < 0.005 and at *p* < 0.001, 2-tailed); Cronbach’s α and McDonald’s ω as scale-reliability coefficients; a *t*-test to explore differences in perceived distress relating to chronic disease carriers and the presence of cohabitants at risk; an ANOVA univariate test with post hoc Tukey HSD and *p* < 0.05 to explore significance between perceived discomfort, gender, and housing specifications; Cohen’s *d* and eta-squared as measures of effect size; normality of the data was assessed by Shapiro–Wilk’s and Levene’s tests; regression assumptions were assessed using Mardia’s multivariate kurtosis index, variance inflation factor (VIF) values, and the Durbin–Watson test; confirmatory factor analysis (CFA) to verify the factor structure of the observed variables and the goodness of fit of the indices as Chi-squared, *p*-values, root-mean-squared error of approximation (RMSEA), comparative fit index (CFI), Tucker–Lewis index (TLI); and structural equation modeling (SEM) analysis to explore the predictors’ effects on self-efficacy and perceived distress. In order to test the adequacy of the model, the following indices were considered: Chi-squared; the relationship between the value of the Chi-squared and the degrees of freedom; goodness-of-fit index (GFI); adjusted goodness-of-fit index (AGFI); root-mean-squared error of approximation (RMSEA); root-mean-squared residual (RMSR); Tucker–Lewis index (TLI); comparative fit index (CFI); normed fit index (NFI); parsimony adjustment to NFI (PNFI); parsimony adjustment to CFI (PCFI); and PCLOSE, to test the null hypothesis that the population RMSEA is no greater than 0.05. The corresponding adequate fit values for the indices were as follows: GFI/TLI/NFI > 0.95; AGFI/CFI > 0.90; RMSEA/RMSR < 0.08; PNFI/PCFI > 0.50; and *p*-value > 0.05.

## 4. Results

### 4.1. Descriptive Analysis

The participants were predominantly residents of small towns with up to 5000 inhabitants (58.6%) and only 6.2% lived in cities with more than 100,000 inhabitants, while 35.2% lived in towns with less than 50,000 inhabitants. The majority of the participants were female, and they spent the lockdown period with an average of 2 other family members, mostly in isolated houses (41.5%) or in flats of 80–100 m^2^ (33.3%). A total of 11% were disabled while 25% had vulnerable and at-risk relatives (e.g., elderly, chronically ill) in their homes. The average age of the participants was 32, with a minimum of 18 and a maximum of 72. Table 2 shows the descriptive values of the variables used in the study.

As shown in Table 3, where the bivariate correlations between the study’s measures are indicated, we observed the inverse relationships between age and the variables of distress (−0.171 **), internal concern (−0.360 **), as well as the fear/closure appraisal (−0.284 **). This suggested that the psychological burden of confinement in the home was more markedly perceived in younger people, in which an attitude oriented toward closure and fear prevailed in response to the negative thoughts prompted by the restrictions. The table also shows a significant inverse association between perceived distress and self-efficacy (−0.239 **), learning/awareness appraisal (−0.133 **), and solidarity/openness appraisal (−0.170 **). However, perceived distress was found to be positively associated with worries intensity (internal concern: 0.409 **; external concern: 0.374 **) and fear/closure-oriented appraisal (0.445). A slight increase in perceived distress was also reported as the number of household members increased (0.120 **).

The *t*-test analyses reported in Table 4 indicated a greater perceived distress in people with chronic illnesses, as well as in those living with vulnerable and at-risk people, such as the elderly and chronically ill. Table 5 reported differences concerning gender and the housing of the participants. A greater perceived distress was shown in females, which was associated with the number of cohabitants but not significantly affected by the type of the housing. Single persons or those who lived in households with three or more cohabitants reported higher values of perceived distress than those found in couples.

### 4.2. A Model of Perceived Self-Efficacy and Distress during COVID-19 Lockdowns

A structural equation modeling (SEM) analysis was subsequently performed by combining the variables of the sources of concern (internal and external), the appraisal patterns of the person (fear/closure, learning/awareness, solidarity/openness), the perception of self-efficacy, and the overall distress during the lockdown conditions, in one explanatory model. The hypotheses were that concerns could activate different appraisal patterns, that these could influence perceptions of self-efficacy, and that, ultimately, the latter would moderate people’s perceived distress. Before conducting the SEM, the following preparatory data analysis was performed. The outliers were assessed by an inspection of a boxplot while normality was assessed using Shapiro–Wilk’s test and the homogeneity of variances was assessed by Levene’s test. There were no outliers, the residuals were normally distributed (*p* > 0.05), and there was a homogeneity of variances (*p* > 0.05). The preliminary verifications of the regression assumptions excluded the presence of multivariate outliers. Mardia’s multivariate kurtosis index (71.63) was below the critical value (*p* (*p* + 2) = 80); therefore, the relationship between the variables could be considered substantially linear. Low co-linearity was indicated by low VIF values at 2.23. For verification of the assumptions on the residuals, the average between the standardized and raw residuals was equal to 0; the Durbin–Watson test had a value of 1.78 and was indicative of the absence of any autocorrelation. As suggested in the literature [59], before conducting a SEM analysis, a separate CFA was performed for each of the constructs: concerns (χ^2^ = 70.824; CFI = 0.971; TLI = 0.953; RMSEA = 0.053), cognitive appraisals (χ^2^ = 101.504; CFI = 0.974; TLI = 0.960; RMSEA = 0.050), general self-efficacy (χ^2^ = 90.992; CFI = 0.972; TLI = 0.962; RMSEA = 0.057), and perceived distress (χ^2^ = 10.528; CFI = 0.992; TLI = 0.985; RMSEA = 0.045). Subsequently, a general SEM analysis was carried out. The tested model showed overall acceptable fit measurements: χ^2^ = 628.09 DF = 355 *p* = 0.000, CMIN/DF = 1.769, RMSR = 0.048, GFI = 0.928, and AGFI = 0.912; including the baseline comparisons: NFI = 0.903, TLI = 0.955, and CFI = 0.955; and the parsimony-adjusted measures: PNFI = 0.789, PCFI = 0.835, RMSEA: 0.038, PCLOSE: 0.999, and RMSEA 90% (0.033; 0.042).

**Figure 2:**
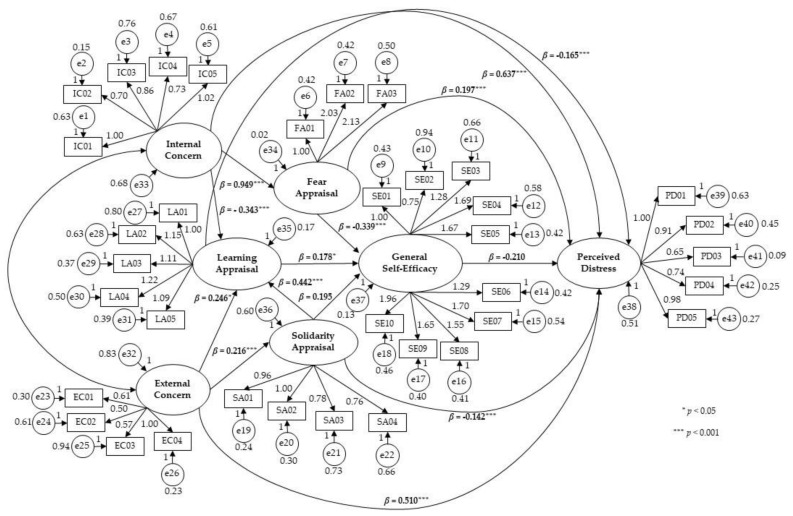
Structural Equation Model.

The model is displayed in Figure 2, which shows that the internal concern activated a prevalent response of fear (a standardized estimate of the regression weight of 0.949 for *p* < 0.001) and, at the same time, limited the learning/awareness response (the standardized estimate of the regression weight of −0.343 for *p* < 0.001). The external concern activated both a learning/awareness response (the standardized estimate of the regression weight of 0.246 for *p* < 0.005) and a response of solidarity/openness and support towards others (the standardized estimate of the regression weight of 0.216 for *p* < 0.001). The appraisal pattern of fear/closure negatively influenced the perception of self-efficacy (the standardized estimate of the regression weight of −0.339 for *p* < 0.001). Predictors of positive effect on self-efficacy were the learning/awareness appraisal (the standardized estimate of the regression weight of 0.178 for *p* < 0.005) and that of solidarity/openness (the standardized estimate of the regression weight of 0.195 for *p* < 0.005). A reinforcing effect of the solidarity/openness appraisal on the learning/solidarity appraisal also emerged (the standardized estimate of the regression weight of 0.442 for *p* < 0.001). Consistent with the hypotheses, there was a direct influence of the concerns on the participants’ perceived distress (internal concern: standardized estimate of the regression weight of 0.637 for *p* < 0.001; external concern: standardized estimate of the regression weight of 0.510 for *p* < 0.001), a moderating effect of distress by self-efficacy (the standardized estimate of the regression weight of −0.210 for *p* < 0.001), by the appraisals of learning/awareness (the standardized estimate of the regression weight of −0.165 for *p* < 0.001), and solidarity/openness (the standardized estimate of the regression weight of −0.142 for *p* < 0.001). However, the fear/closure appraisal showed that it could further potentiate the perception of distress (the standardized estimate of the regression weight of 0.197 for *p* < 0.001).

Table 6 summarizes the maximum likelihood estimates and standardized regression weights estimates.

## 5. Discussion

The SEM analysis confirmed the hypotheses of the study. The direct effect of the concerns on perceived distress was evident, but contextually, it should be noted that the individual’s perceived self-efficacy could positively moderate the level of distress, as has already been noted in the literature. Of particular interest was the role of the type of cognitive appraisal chosen to interpret and control the activations an individual received from the sources of concern. In this case, we also distinguished internal sources (negative emotional experiences such as anxiety, boredom, agitation, tension, pessimism) from external sources (concerns associated with the identification of economic difficulties, interference with future plans, difficulties in the provision of basic necessities, problems in accessing care services, and signs of a general crisis for the entire target community). The model showed that the type of prevailing concern could produce differential effects on the available appraisal models. Internal worries could interact with a threat appraisal, which directed the person towards a fear-and-closure response, which could substantially contribute to increasing the individual’s overall state of distress. The individuals, infected by emotions related to fear and anger, accumulated food, drugs and information, including fake news, they got angry, did not make themself available to help anyone and considered anyone outside home a potential plague-spreader to be accused and/or denounced if seen violating the enforced rules. Several studies highlighted the significant and lasting impact of personal limitations due to the pandemic condition on wellbeing and the mental health of individuals of all age groups, but also especially in young people. More specifically, an increase in stress, worry, insomnia, disorientation, pessimism, obsessive-compulsive symptoms and even gambling disorders has been reported [60,61,62,63,64,65,66,67,68].

Instead of a dysfunctional adaptation of neurotic closure and fear, a more functionally oriented appraisal of coping with the problem could be activated as an alternative. With reference to the well-known distinction between threat and coping appraisals, we distinguished a coping model more focused on the problem and less so on the management of emotional states. While overwhelmed by internal concerns, people could be inclined to have a more conscious approach to the situation by activating functional and concrete action plans. This could also be described as an adaptive learning-oriented assessment through which the person becomes aware of the situation, considers behaviors and adaptions for this new condition, identifies a daily goal, plans future goals commensurate with the new situation, and remains aware of their emotions. The model tested in this study showed that people who had been triggered by external concerns employed either the learning-oriented appraisal (previously described) or an additional functional appraisal model oriented towards growth and supporting others during the emergency, which was a solidarity-based type of openness that encouraged active intervention. They seek out ways to be useful to others while discovering a new way of adapting to change and becoming more self-empathetic [69,70,71]. Based on the observation of the model, while the response of fear and neurotic closure negatively affected perceived self-efficacy, the other two appraisals (adaptive and supportive) contributed significantly to nourishing the individual’s sense of self-efficacy. Openness to others showed a direct effect on self-efficacy that also provided an indirect effect by mediating their level of awareness. In this pattern, it was possible to identify an opportunity for personal growth by not withdrawing into their anxieties but, instead, externalizing their attention via consistent pro-social and networking initiatives, which then enabled them to screen relevant information and plan appropriately for the present situation.

An interesting observation was the reinforcement by the solidarity/openness appraisal on the learning/awareness appraisal. This indicated that the action-oriented appraisal also prompted further learning and the acquisition of greater personal awareness in coping with stressful situations. Overall, the study model showed that the activation of functional appraisals played a significant role in both the direct effect on self-efficacy and also indirectly by helping to contain the level of perceived distress. However, the activation of the appraisal style was based on both the individual’s disposition and experiential modeling [72,73,74,75,76]. For the purposes of prevention and distress containment, it would be appropriate to enhance and promote psycho-educational interventions that urge the adoption of appraisal strategies that would be functional and beneficial for perceived self-efficacy at all developmental stages of the life cycle, including in the elderly and in those who are chronically or acutely ill [77,78,79,80,81].

Several studies on home–life organization during lockdowns have shown the benefits of an active response and daily planning, which not only promoted a reduction in anxiety, boredom, stress, and the perceived overall discomfort, but also contributed to the perceived self-efficacy and resilience for managing the emergency [82,83,84,85]. The promotion active daily management and future planning leads to a relevant protective and preventive approach. Therefore, it would be desirable for educational agencies, health care facilities, as well as local and national institutions to promote the establishment of new services for improved psychological support with a focus on increasing personal and community self-efficacy.

The descriptive analyses of the study highlighted the most vulnerable groups affected by distress during lockdowns: younger people; people with chronic illness; those living with elderly and people with chronic illnesses; women with a particular burden of domestic duties during lockdown; people living in overcrowded settings; and those living alone. Based on the findings of the study, developing a plan for preventative and supportive actions when restrictions are needed should be designed for these vulnerable groups to assist them in internalizing adaptive and functional appraisals and enhancing their self-efficacy. This would enable them to overcome pandemic distress and to increase their coping and resilience in order to overcome future issues.

## 6. Study Limitations

The cross-sectional design of this study limited the predictive generalization of the results. Moreover, the study did not consider variables of socio-economic stratification that could lead to different subjective perceptions of risk and the related anxieties based on socio-economic status [86]. A further limitation of the study was that the participants were predominantly female and residents in a non-urban area. Therefore, including a population living in a large city could have shown different effects on the psychological perceptions of discomfort, such as smaller living spaces being even more common, the presence of more people cohabiting in small spaces, and the reduced availability of outdoor areas, such as balconies, gardens, and courtyards. Considering that the practice of sports was reported as a positive factor for several physical and psychological health outcomes from children and adolescents to the elderly [87,88,89,90,91], future analyses could include a specific assessment concerning the impact of these factors.

## 7. Conclusions

Overall, the results of the study confirmed that during the isolation period, the impact of worries on individual levels of distress was significantly moderated by both the cognitive appraisals employed and the level of perceived self-efficacy. Functional coping appraisals oriented toward learning and solidarity appeared to improve self-efficacy and limit the level of distress, both directly and indirectly. The activation of the threat appraisal was dysfunctional and directed the person towards neurotic closure and withdrawal into their fear and anxiety, lowering their level of self-efficacy and significantly increasing their distress.

## Figures and Tables

**Table 1:** Sample characteristics.

Variable		n (%)
Gender	male	150 (27.6%)
	female	389 (71.4%)
	It was preferred not to declare this	5 (1.0%)
Residence	city (more than 100,000 inhabitants)	34 (6.2%)
	town (less than 50,000 inhabitants)	191 (35.2%)
	small town (up to 5000 inhabitants)	319 (58.6%)
Housing specifications	flat < 60 m^2^	23 (4.23%)
	flat up to 60 m^2^	49 (9.01%)
	flat up to 80 m^2^	78 (14.34%)
	flat up to 100 m^2^	103 (18.93%)
	flat > 100 m^2^	65 (11.95%)
	detached house	226 (41.54%)
Number of cohabitants	alone	38 (6.90%)
	couple	83 (15.60%)
	three	246 (45.22%)
	four	122 (22.43%)
	five	36 (6.62%)
	more than five	19 (3.49%)
Chronic disease carriers		59 (10.85%)
Presence of people at risk at home		131 (24.80%)
Prevailing activities at home	phone calls and/or video calls	122 (22.43%)
	online gaming	22 (4.04%)
	small household tasks	219 (40.26%)
	TV series and movies	115 (21.14%)
	Reading entertainment books	66 (12.13%)
Sports practice	Sports practice at home during lockdown	334 (61.40%)
	Sports practice before COVID-19	323 (59.37%)

**Table 2:** Descriptive statistics.

	Skewness	SE	Kurtosis	SE	Mean	SD
Age	1.190	0.11	0.625	0.21	31.59	11.61
Number of cohabitants	0.279	0.11	0.397	0.21	3.17	1.10
Internal concern	0.363	0.11	−0.141	0.21	2.79	0.80
External concern	0.727	0.11	−0.668	0.21	3.39	0.83
Fear/closure appraisal	0.727	0.11	0.016	0.21	2.19	0.88
Learning/awareness appraisal	−0.067	0.11	0.371	0.21	3.48	0.64
Solidarity/openness appraisal	0.260	0.11	0.366	0.21	3.26	0.69
General self-efficacy	−0.567	0.11	0.984	0.21	3.75	0.65
Perceived distress	0.256	0.11	−0.567	0.21	2.93	0.74

SE = Standard Error; SD = Standard Deviation.

**Table 3:** Bivariate correlations.

	1	2	3	4	5	6	7	8	9
Age	1								
Number of cohabitants	−0.268 **	1							
Internal concern	−0.360 **	0.179 **	1						
External concern	−0.216 **	0.175 **	0.372 **	1					
Fear/closure app.	−0.284 **	0.162 **	0.433 **	0.331 **	1				
Learning/awareness app.	−0.034	−0.049	−0.130 **	−0.097 *	−0.122 **	1			
Solidarity/openness app.	−0.012	0.002	−0.097 *	0.117 **	0.125 **	0.434 **	1		
General self-efficacy	0.102 *	−0.066	−0.267 **	−0.050	−0.266 **	0.275 **	0.304 **	1	
Perceived distress	−0.171 **	0.120 **	0.409 **	0.374 **	0.445 **	−0.133 **	−0.170 **	−0.239 **	1

N = 544; **. Correlation is significant at the 0.01 level (2-tailed). *. Correlation is significant at the 0.05 level (2-tailed). For Age and Number of Cohabitants Spearman’s correlation has been used. App. = Appraisal.

**Table 4:** Differences in perceived distress with respect to direct or indirect contact with the chronic disease.

	M	SD	t	C.I. 95%	*p*	d
People without chronic illness	2.94	0.72	−2.312	(−0.381; −0.123)	<0.05	0.32
People with chronic illness	3.25	0.91				
Living without elderly or chronically ill	2.79	0.91	−2.782	(−0.312; −0.153)	<0.005	0.35
Living with elderly or chronically ill	3.41	0.87				

**Table 5:** Differences in perceived distress in relation to gender, the size of the house, and the number of cohabitants.

		M	SD	F	*p*	η^2^
Gender	Male	2.70	0.93	7.58	<0.05	0.03
	Female	3.02	0.84			
	Prefers not to answer	2.67	0.53			
Extent of the housing	flat < 60 m^2^	2.70	0.79	1.437	>0.05	0.01
	flat up to 60 m^2^	2.76	0.87			
	flat up to 80 m^2^	2.96	0.83			
	flat up to 100 m^2^	3.09	0.91			
	flat > 100 m^2^	2.87	0.87			
	detached house	2.93	0.89			
Number of cohabitants	alone	2.81	0.88	2.911	<0.05	0.03
	couple	2.63	0.82			
	three	2.97	0.88			
	four	3.04	0.90			
	five	3.08	0.80			
	more than five	3.10	0.92			

**Table 6:** Maximum likelihood estimates and standardized regression weight estimates.

Label		Label	Estimate	S.E.	C.R.	*p*	SWE
Internal Concern	→	Fear/Closure Appraisal	0.506	0.046	11.091	***	0.949
Internal Concern	→	Learning/Awareness Appraisal	−0.203	0.048	−4.232	***	−0.343
External Concern	→	Learning/Awareness Appraisal	0.132	0.045	2.910	0.004	0.246
External Concern	→	Solidarity/Openness Appraisal	0.188	0.048	3.909	***	0.216
Solidarity/Openness Appraisal	→	Learning/Awareness Appraisal	0.478	0.042	6.511	***	0.442
Fear/Closure Appraisal	→	General Self-Efficacy	−0.316	0.055	−5.784	***	−0.339
Learning/Awareness Appraisal	→	General Self-Efficacy	0.151	0.053	2.866	0.004	0.178
Solidarity/Openness Appraisal	→	General Self-Efficacy	0.101	0.031	3.278	0.001	0.195
General Self-Efficacy	→	Perceived Distress	−0.174	0.039	−4.134	***	−0.210
Fear/Closure Appraisal	→	Perceived Distress	0.170	0.041	4.416	***	0.197
Internal Concern	→	Perceived Distress	0.592	0.044	13.383	***	0.637
External Concern	→	Perceived Distress	0.478	0.034	12.881	***	0.510
Learning/Awareness Appraisal	→	Perceived Distress	−0.225	0.042	5.105	***	−0.165
Solidarity/Openness Appraisal	→	Perceived Distress	−0.180	0.039	−4.567	***	−0.142

MLE, Maximum Likelihood Estimates; SWE, Standardized Regression Weight Estimates. *** *p* < 0.001.

## Data Availability

The data presented in this study are available on request from the corresponding author.
